# Integrated UPLC-Q-TOF-MS/MS and Network Pharmacology Approach to Investigating the Metabolic Profile of Marein of *Coreopsis tinctoria* Nutt.

**DOI:** 10.1155/2022/6707811

**Published:** 2022-05-23

**Authors:** Jing Liu, Xuejing Cheng, Xin Zheng, Yumeng Shi, Chunxia Li, Qiaoyu He, Yue Li, Xiaopeng Chen

**Affiliations:** ^1^State Key Laboratory of Component-Based Chinese Medicine, Institute of Traditional Chinese Medicine, Tianjin University of Traditional Chinese Medicine, Tianjin 301617, China; ^2^Beijing Analytical Center-SSL Shimadzu (China) Co., LTD, Beijing 100020, China

## Abstract

Marein is the main active compound of *Coreopsis tinctoria* Nutt., and its main activities include antioxidant, hypoglycemic, and hypotensive. After oral administration of marein, the blood concentration of marein is low. The metabolites of marein have not been reported systematically. In this study, a rapid and systematic method based on ultra-high performance liquid chromatography-quadrupole time-of-flight mass spectrometry (UPLC-Q-TOF-MS/MS) was established to detect metabolites of marein *in vivo* (plasma and urine) after oral administration and injection. Sixty-one metabolites were identified. The metabolites are formed through a wide range of metabolic reactions, including hydroxylation, glucuronidation, methylation, hydrolysis, and desorption of hydrogen. The liver microsome incubation was further used to investigate the metabolic rate of marein. Network pharmacology was applied to study the targets and pathways of marein and its metabolites. Marein and its metabolites act on the same targets to enhance the therapeutic effect. This research illuminates the metabolites and metabolic reaction of marein and establishes a basis for the development and rational utilization of *C. tinctoria*. Meanwhile, the analysis of prototype and metabolites together by network pharmacology techniques could provide a methodology for the study of component activity.

## 1. Introduction

*Coreopsis tinctoria* Nutt. (*C. tinctoria*), also known as “snow chrysanthemum,” is native to North America and is currently grown worldwide [1, 2]. It is a tea material used to deal with diabetes [3], hyperlipidemia, and hypertension [4–9]. North American Indians used dried *C. tinctoria* tea to treat internal organ pain and bleeding. *C. tinctoria* is used to treat diabetes in Portugal [10–12]. Pharmacological research shows that the main components of the *C. tinctoria* are flavonoids with a variety of biological activities [13–15].

Marein, the glucoside of chalcone, is the main active component of flavonoids in *C. tinctoria* [16–18]. It has effects on lowering blood sugar, regulating blood lipids, and anti-hypertension [3]. Experiments *in vitro* have proved that marein can resist oxidation [19, 20], reduce triacylglycerol content, and have a certain protective effect on pancreatic islet cells MIN6 [11]. Marein also improves glucose metabolism disorder induced by high glucose in HepG2 cells, which could significantly prevent insulin resistance induced by high glucose [21, 22]. And marein can improve the abnormal glucose and lipid metabolism of hypertrophic H9c2 cells by reducing the expression of HIF-1*α* [23]. It also prevents diabetic nephropathy by inhibiting the expression of SGLT2 in the kidney [24].

However, after healthy volunteers drank a tea of *C. tinctoria*, no marein is detected in their blood [21]. In the preliminary experiments of our team, after intragastric administration in rats, no marein was found in plasma. The low blood concentration and poor bioavailability of marein make it difficult to explain the remarkable curative effect. Therefore, a study of the metabolic processes and pharmacological action of metabolites of marein are needed.

After oral administration, natural products take effects *in vivo* based on the combination of prototype and metabolites. Network pharmacology, as a powerful tool for analysis of multicomponent effects [25, 26], reveals the complex biological network relationships among compounds, genes, and targets by using network visualization techniques to analyze the behavior of compounds acting on different targets, cells, and organs at the molecular and gene levels. Using the network pharmacology method, the “compound-target-pathway” network can be constructed according to the structure and efficacy of compounds, which can effectively predict the activities and mechanism of components.

In this article, a specific and sensitive ultra-high performance liquid chromatography-quadrupole time-of-flight mass spectrometry (UPLC-Q-TOF-MS/MS) method was established for rapid screening and systematic identification of metabolites of marein. The oral and injected metabolites of marine were identified in biological samples including plasma and urine. Then, an *in vitro* incubation system of rat liver microsomes was established. Network pharmacology was used to study the component-target-pathway network of marein and its metabolites. The research establishes a scientific basis for the development and rational use of *C. tinctoria*.

## 2. Materials and Methods

### 2.1. Chemicals, Reagents, and Materials

Marein (batch number: 20190528, purity ≥99%) was isolated from *C. tinctoria* in the laboratory. The reference standard (purity >98%) of marein (batch number: Y31M9H57751) was purchased from Shanghai YuanYe Biotechnology Co. Ltd. (Shanghai, China). Rat liver microsome and *β*-nicotinamide adenine dinucleotide phosphate (NADPH) were obtained from PrimeTox (Wuhan, China). HPLC-grade formic acid was supplied from ROE (St. Louis, MO, USA). Methanol and acetonitrile of MS grade were provided by Sigma-Aldrich Co. (St, Louis, USA). The ultrapure water was purified with a Milli-Q water purification system (Millipore, Milford, MA, USA).

### 2.2. Animal Handling and Specimen Collection

Forty-eight healthy male Sprague–Dawley rats, SPF grade, weighing 250 ± 10 g, were purchased from Huafukang Laboratory Animal Technology Co. Ltd. (Beijing, China). Before use, all animals were reared for 5 days at a constant temperature of 25°C, a humidity of 40–60%, and a 12-hour light-dark cycle. All experimental protocols strictly followed the Guide for the Care and Use of Laboratory Animals (NIH Publication No. 8023, revised 1978), the Guidance Suggestions for the Care and Use of Laboratory Animals issued by the Ministry of Science and Technology of China (2006), and the Animal Use and Care Committee of Tianjin University of Traditional Chinese Medicine (No. IRMDWLL-201903).

#### 2.2.1. Oral Administration

After adaptive feeding for 1 week, 24 SD rats were randomly divided into 4 groups: blank blood sample group, blank urine group, marein blood sample group, and marein urine group. The experimental animals were fasted 12 hours before the administration and free drinking water. Marein was dissolved with an appropriate amount of CMC-Na solution and was administered to SD rats by gavage at a dose of 200 mg/kg. The blank blood sample group was given an equal volume of CMC-Na solution.

#### 2.2.2. Administration by Injection

After adaptive feeding of 24 SD rats for 1 week, they were randomly divided into 4 groups, namely: blank blood sample group, blank urine group, marein blood sample group, and marein urine group. The experimental animals were fasted 12 hours before the administration and free drinking water. Marein was dissolved in appropriate amounts of saline and administered to SD rats via tail vein injection at a dose of 10 mg/kg. The blank group was injected with an equal volume of saline through the tail vein.

#### 2.2.3. Plasma Sample Collection

Blood samples were collected from the fundus venous plexus of rats at 0, 0.08, 0.25, 0.5, and 1 h before and after gavage and injection. The blood samples were centrifuged (9,900 g, 10 min) to obtain plasma samples. All plasma samples were stored at −80°C until they were processed and analyzed.

#### 2.2.4. Urine Samples Collection

Urine samples were collected for 24 hours by gavage and injection in a metabolic cage. All urine samples were stored at −80°C until they were processed and analyzed.

### 2.3. Sample Preparation

Take 100 *μ*L of plasma from each group at the same time point and combine, add 900 *μ*L of acetonitrile, vortex for 3 min, and centrifuge (4°C, 19,500 g) for 10 min. Take the supernatant, blow-dry, and concentrate and then add 900 *μ*L of acetonitrile. Vortex for 3 minutes and then centrifuge (4°C, 19,500 g) for 10 minutes. Take the supernatant, dry it with nitrogen, and concentrate. Add 100 *μ*L of 50% methanol to reconstitute the residue (methanol:water = 1:1), vortex, and centrifuge (4°C, 19,500 g) for 10 minutes. The supernatant was taken and analyzed by UPLC-Q-TOF-MS/MS. The blank plasma sample was processed in the same way.

Take 400 *μ*L of urine, add 800 *μ*L of methanol, vortex for 3 minutes, and centrifuge (4°C, 19,500 g) for 10 minutes. Take the supernatant, blow-dry, and concentrate and then add 800 *μ*L of methanol to reconstitute, vortex for 3 minutes, and centrifuge (4°C, 19,500 g) for 10 minutes. In parallel, the supernatants were combined, dried, concentrated with nitrogen, and finally reconstituted with 100 *μ*L 50% methanol (methanol:water = 1:1). The supernatant was analyzed by UPLC-Q-TOF-MS/MS. The blank urine sample was processed in the same way.

### 2.4. Metabolism in Rat Liver Microsomes

The total volume of the incubation system is 500 *μ*L, including phosphate buffer (0.1 M, pH 7.4) 470 *μ*L, and NADPH generating system 30 *μ*L (Solution A: 25 *μ*L and Solution B: 5 *μ*L). The process is completed on an ice bath.

Marein blank control group: Take the above incubation system, add 5 *μ*L of aqueous solution to an ice bath, incubate at 37°C for 5 min, and then add 15 *μ*L of liver microsomes to the ice bath. After gently mixing the incubation system by hand, start the incubation at 37°C in a water bath. The incubation time is 0, 5, 15, 30, 45, 60, 90, and 120 min, and each sample is paralleled 3 times. When the reaction is complete, stop the reaction with the same volume of ice acetonitrile to completely precipitate the protein, centrifuge at 4,200 g for 10 min, aspirate the supernatant and centrifuge at 19,500 g for 10 min, and inject 2 *μ*L for analysis.

Marein inactivated liver microsomes group: First inactivate liver microsomes, take the incubation system, add 5 *μ*L of the marein reference substance stock solution on an ice bath, incubate at 37°C for 5 min, and then add 15 *μ*L of inactivation to the ice bath. The following procedures were similar to the blank control group.

Marein liver microsomes group: Take the incubation system, add 5 *μ*L of the marein reference substance stock solution to an ice bath, incubate at 37°C for 5 min, and then add 15 *μ*L of liver microsomes to the ice bath. The following operation was the same as the blank control group.

### 2.5. UPLC-MS/MS Conditions

Used UPLC-Q-TOF-MS/MS 9030 system (Shimadzu, Japan), chromatography column: Shim-pack GISS C18 column (100 × 2.1 mm, 1.9 *μ*m). Mobile phase: A phase 0.1% formic acid water, B phase acetonitrile, gradient elution, elution gradient: 0∼1 min, 5∼10% B; 1∼2 min, 10% B; 2∼3 min, 10∼15% B; 3∼5 min, 15∼20% B; 5∼11 min, 20∼40% B; 11∼13 min, 40∼80% B; 13∼15 min, 80∼95% B; 15∼17 min, 95% B; 17∼20 min, 5% B. Flow rate: 0.3 mL/min; column temperature: 40°C.

MS operation conditions were as follows. The Q-TOF-MS spectrometer was configured with an electrospray ion source (ESI) operating in the negative and positive ion modes. Ion source interface voltage is −3.0 Kv. Nitrogen is used as the drying gas and the atomizing gas. The drying gas flow rate is set to 10 L/min, and the atomizing gas flow rate is set to 3.0 L/min. Air is used as heating gas, and the flow rate is set to 10 L/min; argon is used as collision gas; desolvent tube temperature is 250°C; heating block temperature is 400°C; and interface temperature is 300°C. Scan modes are MS Scan (*m/z* 100–500; 500–1,000) and MS/MS (*m/z* 50–500; 50–1,000). The collision energy (CE) is 35 ± 17 V.

In the study of metabolism *in vivo*, the injection volume was 5 *μ*L, while that *in vitro* metabolism was 2 *μ*L.

### 2.6. Data Analysis

The data obtained by UPLC-Q-TOF-MS/MS were analyzed with Formula Predictor Server software. Target fragments are analyzed by ACD/Labs software accurate mass calculator (Shimadzu City, Japan).

In the liver microsomes study, the 0 min peak area of marein was regarded as 100%, and other time points were compared with the 0 min peak area to obtain the remaining percentage (area%). According to the natural logarithm of area% and incubation, the time slope *k* was obtained by linear regression. According to formula (1), the half-life t_1/2_/min of marein in liver microsomes could be obtained. According to formulas (2)–(5), the half-lives in liver microsomes, intrinsic clearance rate CL_int_, *in vivo* intrinsic clearance rate CL′_int_, liver clearance rate CL_*H*_, and liver extraction rate ER were calculated.

(1) $$\begin{array}{c} {\text{t}}_{1/2}=\frac{-0.693}{k}, \end{array}$$

(2) $$\begin{array}{c} {\text{CL}}_{\mathrm{int}}=\frac{0.693}{{\text{t}}_{1/2}}\frac{\text{incubation}\left(\text{mL}\right)}{\text{microsomes}\left(\text{mg}\right)}, \end{array}$$

(3) $$\begin{array}{c} {\text{CL}}_{\mathrm{int}}^{\prime }=\frac{0.693}{{\text{t}}_{1/2}}\cdot \frac{\text{incubation}\left(\text{mL}\right)}{\text{microsomes}\left(\text{mg}\right)}\cdot \frac{\text{Liver}\left(\text{g}\right)}{BW\left(\text{kg}\right)}\cdot \frac{\text{microsmes}\left(\text{mg}\right)}{\text{Liver}\left(\text{g}\right)}, \end{array}$$

(4) $$\begin{array}{c} {\text{CL}}_{H}=\frac{Q\cdot {\text{CL}}_{\mathrm{int}}^{\prime }}{Q+{\text{CL}}_{\mathrm{int}}^{\prime }}, \end{array}$$

(5) $$\begin{array}{c} \text{ER}=\frac{{\text{CL}}_{H}}{Q}. \end{array}$$

The physical and chemical parameters: The liver tissue per kilogram of body weight for rat (Liver (g)/BW (kg)) was 40 g. *Q* is hepatic blood flow (66 ml/min/kg for rat), and CL′_int_ is the intrinsic clearance. The microsomal protein per gram of liver tissue (microsomes (mg)/liver (g)) was 45 mg.

### 2.7. Network Pharmacology

The Mol2 structural formulas of marein and its metabolites were obtained from the PubChem database (https://pubchem.ncbi.nlm.nih.gov/) and then imported into the PharmMapper database (http://www.lilab-ecust.cn/pharmmapper/) to predict the possible targets of the ingredients [27–29], screened with a normalized fit score greater than 0.5, and then standardized through the Uniprot database (https://www.uniprot.org/) [30]. Select this species as “*Homo sapiens*” and get multiple targets for each chemical component to exert pharmacological effects. The intersection of targets was processed; duplicate values were deleted; and the PPI protein interaction network was constructed by importing the STRING website (https://www.string-db.org/) [31]. Kernel targets were screened by importing Cytoscape software [32]. After that, KEGG (Kyoto Encyclopedia of Genes and Genomes) signaling pathway enrichment and GO (Gene Ontology) analysis were carried out in the Database for Annotation, Visualization, and Integrated Discovery (DAVID; https://david.ncifcrf.gov/summary.jsp) [33]. The major action pathways were screened out to explore the relationship between “component-important target-pathway.”

## 3. Results and Discussion

### 3.1. Identification of Marein Metabolites in Rats after Oral Administration

**Figure 1:**
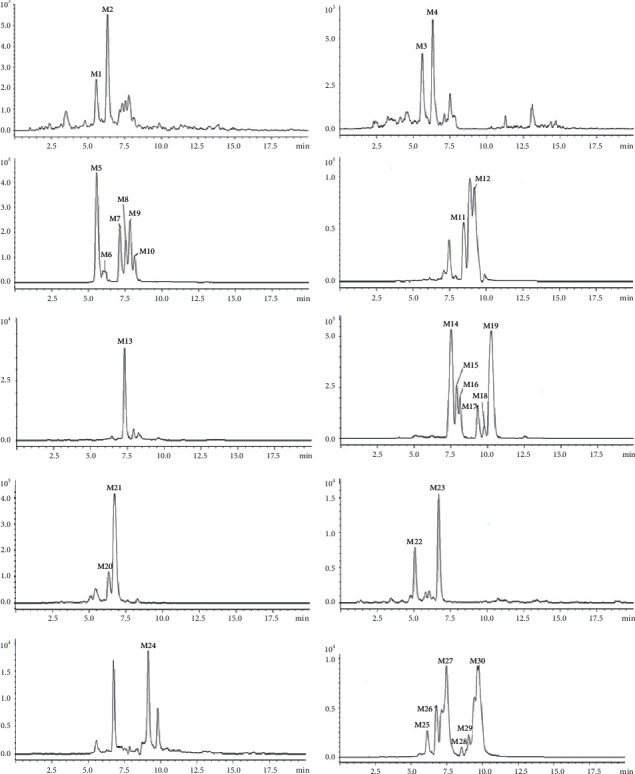
The extracted ion chromatograms of metabolites after oral administration of marein in plasma and urine.

UPLC-Q-TOF-MS/MS was used to study the chromatographic and mass spectrometric cleavage behavior of marein and its metabolites. Thirty metabolic components for oral administration were identified, and 25 and 19 metabolites were detected in plasma and urine separately. The detailed metabolites information after orally administrated of marein was summarized in Table 1, and extracted ion chromatograms of metabolites were shown in Figure 1. The results show that no prototype components were found in the rats' plasma and urine after the rats were given marein by gavage. It means that after intragastric administration, marein is metabolized and mainly exists in the form of metabolites in the blood.

**Figure 2:**
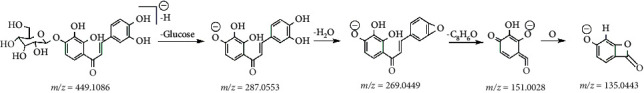
The cleavage of marein.

The MS and MS/MS chromatogram of the marein were recorded. Under the chromatographic conditions used, the molecular formula is C_21_H_22_O_11_, and the retention time is 8.189 min. The parent ion is [M-H]^−^*m/z* = 449.1077, and product ions are *m/z* = 287.0553 [M-H-Glu]^−^, 269.0440 [M-H-Glu-H_2_O]^−^, 151.0025 [M-H-Glu-H_2_O-C_8_H_6_O]^−^, and 135.0441 [M-H-Glu-H_2_O-C_8_H_6_O-O]^−^. The cleavage of marein is shown in Figure 2.

The metabolite M1 is eluted at 5.62 min, and the quasimolecular ion peak [M-H]^−^ is *m/z* 495.0780 in the negative ion mass spectrum, and its molecular formula is predicted to be C_21_H_20_O_14_. In the MS/MS spectrum of M1, the *m/z* of the product ions are 151.0034 [M-H-Glu-H_2_O-C_8_H_4_O_4_]^−^ and 135.0426 [M-H-Glu-H_2_O-C_8_H_4_O_4_-O]^−^. It is consistent with the product ions at 151 and 135 of marein. Combined with the molecular formula, it is speculated that it has 3 more hydroxyl groups. Therefore, it is preliminarily determined to be a dehydrogenation and hydroxylation metabolite.

The retention time of metabolite M2 is 6.37 min. The anion first-order mass spectrometry shows that the excimer ion peak [M-H]^−^ is *m/z* 495.0708, and its molecular formula is predicted to be C_21_H_20_O_14_. Fragment ions *m/z* 301.0352, 151.0022, and 135.0445 can be seen by secondary mass spectrometry. Like M1, it suggests dehydrogenated and hydroxylated metabolites.

The retention time of metabolite M3 is 5.65 min, and the excimer ion peak [M-H]^−^ is *m/z* 479.0831 in negative ion first-order mass spectrometry, and its molecular formula is predicted to be C_21_H_20_O_13_. Characteristic ion fragments *m/z* 269.0442, 151.0031, 135.0438 are generated by secondary scanning lysis. The molecular weight is 16 less than that of M1 and M2. It is judged that there is one less hydroxyl group, and it is speculated that the dehydroxylation products of M1 and M2.

The excimer ion peak [M-H]^−^ of the metabolite M4 eluted at 6.34 min is *m/z* 479.0831, and its molecular formula is predicted to be C_21_H_20_O_13_, which is an isomer with M3. The MS mass spectrum correspondingly produces characteristic fragment ions *m/z* 151.0033 and 135.0438. It is also the dehydrogenation and hydroxylation metabolites of marein.

The excimer ion peak [M-H]^−^ of metabolite M5-M10 is *m/z* 463.0882, eluted at 5.58, 5.99, 7.17, 7.55, 7.85, and 8.13 min, respectively. Its molecular formula is predicted to be C_21_H_20_O_12_. The main secondary fragment ions include *m/z* 301.0709 [M-H-Glu]^−^, 287.0552 [M-H-Glu-O]^−^, 269.0445 [M-H-Glu-O-H_2_O]^−^, 151.0028 [M-H-Glu-O-H_2_O-C_8_H_6_O]^−^, and 135.0444 [M-H-Glu-O-H_2_O-C_8_H_6_O-O]^−^. The molecular weight is 14 higher than that of marein and 16 less than that of M3 and M4. Combined with the predicted molecular formula, both of them are dehydroxylated metabolites of M3 and M4.

The metabolite M11-M12 is an isomer, and its excimer ion peak [M-H]^−^ is *m/z* 479.1195, and the retention time is 8.47 and 9.27 min, respectively. Its molecular formula is predicted to be C_22_H_22_O_12_. The MS-MS information of the metabolite M11 is *m/z* 303.0871 [M-H-Glu-O-CH_3_]^−^, 269.0457 [M-H-Glu-O-CH_3_-H_2_O]^−^, and 151.0028 [M-H-Glu-O-CH_3_-H_2_O-C_8_H_6_O]^−^. The secondary mass spectrum of the metabolite M12 shows fragment ions *m/z* 303.0870, 151.0027, and 135.0443 [M-H-Glu-O-CH_3_-H_2_O-C_8_H_6_O-O]^−^. Therefore, it is identified as hydroxylated and methylated metabolites.

The metabolite M13 is eluted at 7.32 min, and the eximolecular ion peak [M-H]^−^ is *m/z* 447.0932. Its molecular formula is predicted to be C_21_H_20_O_11_. The characteristic fragment ions *m/z* 285.0395 [M-Glu]^−^, 151.0025 [M-Glu-H_2_O-C_8_H_6_O]^−^, and 135.0444 [M-Glu-H_2_O-C_8_H_6_O-O]^−^ are generated by the secondary mass spectrometry. Its molecular weight is 2 less than that of marein, and it may lose 2 hydrogens. So it is presumed to be the dehydrogenation product of marein.

The excimer ion peak [M-H]^−^ of the metabolite M14-M19 is *m/z* 491.1195, eluted at 7.52, 7.94, 8.13, 9.33, 9.77, and 10.26 min, respectively. Its molecular formula is predicted to be C_23_H_24_O_12_. The main secondary fragment ions are *m/z* 315.0865, 269.0443 [M-H-Ac-Glu-H_2_O]^−^, 151.0021 [M-H-Ac-Glu-H_2_O-C_8_H_6_O]^−^, and 135.0441 [M-H-Ac-Glu-H_2_O-C_8_H_6_O-O]^−^. It is determined that M14-M19 are isomers of the acetylated product of marein.

The retention times of the metabolites M20-M21 are 6.36 and 6.74 min, respectively. The anion first-order mass spectrometry shows that the excimer ion peak [M-H]^−^ is *m/z* 641.1359. The molecular formula is predicted to be C_27_H_30_O_18_. Fragment ions *m/z* 465.1017 [M-H-Glu-CH_3_]^−^, 303.0866 [M-H-2Glu-CH_3_-O-H_2_O-C_8_H_6_O]^−^, 151.0028 [M-H-2Glu-CH_3_-O-H_2_O-C_8_H_6_O]^−^, and 135.0442 [M-H-2Glu-CH_3_-O-H_2_O-C_8_H_6_O-O]^−^ are observed by the secondary mass spectrometry, 162 more than the metabolite M11, which is speculated to be the glucose acidification product of M11.

The metabolite M22-M23 excimer ion peak [M-H]^–^ are both *m/z* 625.1410, eluted at 5.07 and 6.73 min, respectively. They are isomers, whose molecular formula is predicted to be C_27_H_30_O_17_. Its molecular weight is 162 higher than that of marein, so it is presumed to be the product of marein glucuronidation. The secondary mass spectra generate the characteristic fragment ions *m/z* 449.1058 [M-H-Glu]^−^, 287.0553[M-H-2Glu]^−^, 269.0461[M-H-2Glu-H_2_O]^−^, 151.0025[M-H-2Glu-H_2_O-C_8_H_6_O]^−^, and 135.0447[M-H-2Glu-H_2_O-C_8_H_6_O-O]^−^.

The metabolite M24 is eluted at 9.15 min, and the eximolecular ion peak [M-H]^−^ is *m/z* 287.0561 in anion first-order mass spectrometry. Its molecular formula is predicted to be C_15_H_12_O_6_. Its relative molecular weight is 162 less than that of marein, suggesting the removal of a molecule of glucose group metabolite. The MS mass spectrum shows the fragment ion *m/z* 135.0447 [M-H_2_O-C_8_H_6_O-O]^−^. It is confirmed that M24 is the product of marein aglycone.

The excimer ion peak [M-H]^−^ of the metabolite M25-M30 is *m/z* 477.1038, and the retention time is 6.16, 6.80, 7.46, 8.56, 9.07, and 9.70 min, respectively. Its molecular formula is predicted to be C_22_H_22_O_12_. The main secondary fragment ions are *m/z* 301 [M-Glu-CH_3_]^−^, 151 [M-Glu-CH_3_-O-H_2_O-C_8_H_6_O]^−^, and 135 [M-Glu-CH_3_-O-H_2_O-C_8_H_6_O-O]^−^. It is 14 more than the metabolites M5-M10, and it is determined that M25-M30 are the methylation products of M5-M10.

### 3.2. Identification of Marein Metabolites in Rats after Injection

The prototype compounds and metabolites in the urine and blood samples of rats after the tail vein injection of marein were analyzed. By comparing the retention time in the chromatograms of the administration group and the blank group to track the metabolites, 31 metabolites were detected in injection, and 23 and 26 were detected in plasma and urine separately.

**Figure 3:**
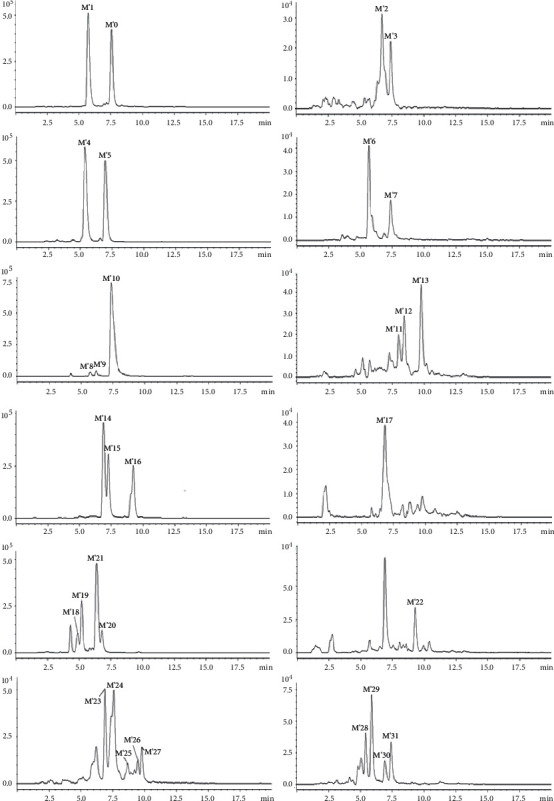
The extracted ion chromatograms of metabolites after injection of marein in plasma and urine.

Among them, M′0 is the prototype marein. And M′1 has the same molecular weight as M′0, which may be presumed to be the hydrogenation product after dehydrogenation. M′2 and M′3 can be inferred to be hydroxylated and sulfated metabolites according to their molecular weight and molecular formula. M′4 and M′5 have a molecular weight of 80 more than that of M′0. M′6 and M′7 are the hydroxylated products after the dehydrogenation of 2 hydrogens. Compared with M′0, the molecular weight of M′8, M′9, and M′10 are 2 less, which is supposed to be the removal of 2 hydrogens. The molecular weight of M′11, M′12, and M′13 are 16 less, so they may be dehydroxylated products. M′14-M′16 are 14 more than M′0 and are methylated products. M′17 is inferred to be the removal of 2 hydroxy groups after the addition of 2 hydrogens. M′18-M′21 is presumed to be a glycosylation metabolite, and M′22 is presumed to be a deglycosylation product and an aglycone of marein. M′23-M′27 may be the methylation products of M′6-M′7. And M′28-M′31 may be the sulfuration products of M′14-M′16. The detailed metabolites information after injection of marein was summarized in Table 2, and extracted ion chromatograms of metabolites were shown in Figure 3.

**Figure 4:**
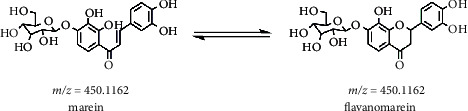
Isomerization of marein and flavanomarein.

More interestingly, the prototype components were detected after the tail vein injection, and isomerization also occurred, that is, the two isomers marein and flavanomarein. The structure was shown in Figure 4. After intravenous administration, the two transformed into each other. It provides a reference for further research on the pharmacological studies of marein (chalcone glycoside) and flavanomarein (dihydroflavonoid glycoside). Regarding the isomerization reaction, it has also been reported in the metabolism of flavonoids with similar structures: rat gavage liquiritigenin (LG; dihydroflavonoids), isoliquiritigenin (ILG; chalcone), liquiritin (LQ; dihydroflavonoid glycoside), and isoliquiritin (ILQ; chalcone glycoside). Its chalcone components could realize the conversion with the corresponding dihydroflavonoids [34].

### 3.3. Oral Administration and Injection Metabolic Pathways Analysis

**Figure 5:**
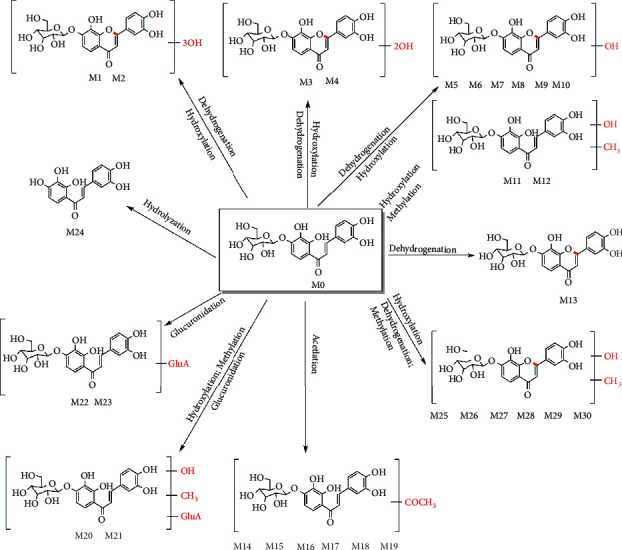
The metabolic pathways of marein after oral administration in plasma and urine.

**Figure 6:**
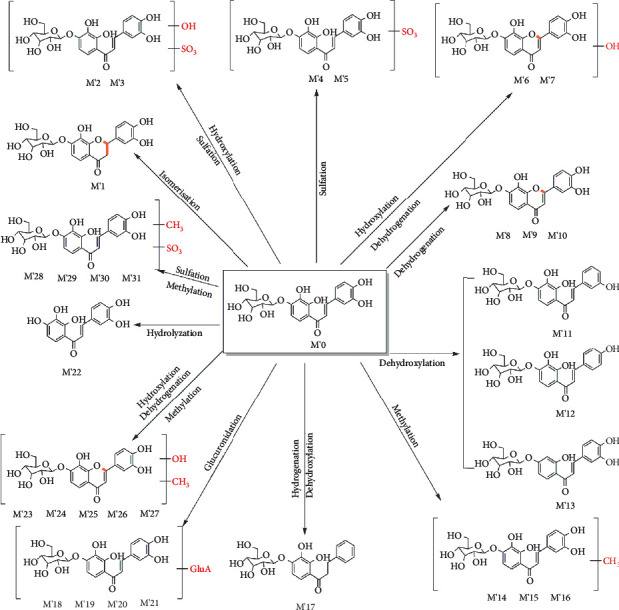
The metabolic pathways of marein after injection of plasma and urine.

The possible metabolic pathways of marein via oral administration and injection were shown in Figures 5 and 6, respectively. The metabolic pathways include sulfation, hydrogenation, hydroxylation, methylation, glucuronidation, hydrolysis, dehydroxylation, isomerization, and dehydrogenation.

*In vivo*, 30 marein metabolites were observed in oral administration, while 31 marein metabolites were observed in injection. Among them, 9 shared metabolites were found in oral and injectable administration, including M5, M13, M22, M23, M24, M26, M27, M28, and M30 in oral administration, which correspond to M′6, M′10, M′18, M′21, M′22, M′23, M′24, M′25, and M′27 in injection administration one by one. And we marked it with ^*∗*^ in Tables 1 and 2.

Marein belongs to chalcone glycoside. According to the results of oral and injection studies, it can be hydrolyzed into its aglycones *in vivo*, and its aglycone M25 (chalcones) could be further metabolized. Studies on the metabolism of components with similar structures have been reported. For example, after intragastric administration of isoliquiritin (chalcone glycoside) and isobavachalcone (chalcones) in rats, metabolic pathways *in vivo* include glucuronidation, hydrolysis, isomerization, sulfation, hydrogenation, hydroxylation, and other metabolic reactions [35]. The metabolic rule of marein is consistent with that of other chalcone glycosides, indicating the common rule of chalcone glycoside metabolism.

By comparing the experimental results of oral and injection of marein, it is found that their metabolic pathways include hydroxylation, glucuronidation, methylation, hydrolysis, and dehydrogenation. The difference is that after oral administration, marein was not detected in the original form, and mainly existed in the form of metabolites in blood. While injection administration, that is, in the absence of gastrointestinal absorption and liver first-pass effect, the prototype component of the glycoside can be detected in the body. It can be seen that the first-pass effect of the gastrointestinal tract and the liver plays an important role in the oral metabolism of marein, which makes the metabolites different between the two administration methods.

### 3.4. Metabolism of Marein in Rat Liver Microsomes

**Figure 7:**
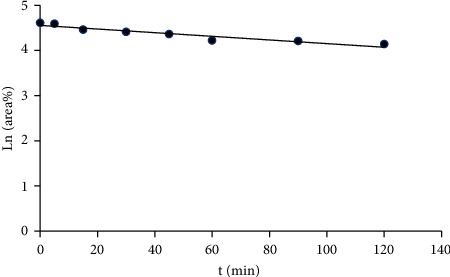
The linear graph of Ln (area%) and the time of incubation about marein in rat liver microsomes.

Liver microsomes can effectively evaluate the influence of metabolic enzymes on compound metabolism. In this experiment, an *in vitro* incubation system for rat liver microsomes was established. The UPLC-Q-TOF-MS/MS method was used to identify the metabolites of marein in liver microsomes and to study the metabolic stability in vitro. The data of Ln (area%) and the time of incubation were shown in Figure 7; the data of area%, Ln (area%), and the time of incubation were shown in Table 3; and the data of the half-time period, CL_int_, CL′_int_, CL_H_, and ER were shown in Table 4. The results show that the *in vitro* metabolic half-life t_1/2_ of marein in liver microsomes is 177.69 min and the liver extraction rate is 0.15. It shows that the metabolic stability of marein in liver microsomes is good, indicating that liver microsomes have no significant effect on the metabolism of marein. Since the prototype components of marein were not detected in the body after intragastric administration, we speculate that liver metabolism is not the main reason for the low bioavailability of marein. According to existing research, the gastrointestinal tract may play a key role in the metabolism of marein. The study of rutin gives us some enlightenment. Rutin can be combined with enzymes in the intestine after oral administration. The enzymes in the intestine hydrolyze most of the rutin into quercetin, and only a small part of the rutin can be absorbed into the blood. Given the similar structure of marein, flavanomarein, and rutin, it is speculated that after oral administration of marein in rats; the enzymes in the intestinal tract will hydrolyze marein; and its metabolites are absorbed into the blood.

### 3.5. Network and Target Biological Function Analysis

**Figure 8:**
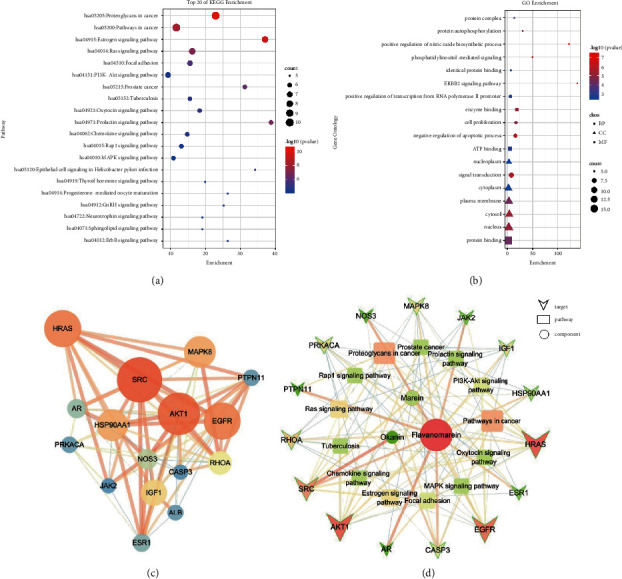
Network pharmacology analysis of three components of *Coreopsis tinctoria* Nutt. KEGG pathway enrichment results (a), GO enrichment results (b), PPI results (c), and marein-related components—key targets—action pathway network (d).

Predictive analysis of the related targets is based on marein and its metabolites: okanin (marein's aglycon, M′1) and flavanomarein (the isomer of marein, M24 or M′22). Two hundred and sixty-five targets were obtained. Then input the obtained targets into STRING to predict the interaction relationship between protein and protein. The species was selected as “*Homo sapiens*,” and set a score greater than 0.700 as the basis for high confidence in the interaction, hiding the disconnected nodes. Export the tsv format file and import it into Cytoscape 3.6.1 for network visualization and topological attributes analysis. The higher the degrees of nodes are, the more important the nodes are to maintain the stability of the entire network. We sorted the degree values and selected the core targets with the corresponding degree values greater than four times the median value as the potential key targets related to activity and visualized them (Figure 8(c)). The nodes with higher degrees are in a brighter color, and the node size and edge size represent the betweenness centrality and edge betweenness of the nodes, respectively. The top 5 core targets are SRC, AKT1, EGFR, HRAS, and HSP90AA1. KEGG signaling pathway enrichment and GO function analysis were performed on 16 targets with degree values greater than quadruple the median as the core targets by using the Functional Annotation tool in the DAVID database.

In Figure 8(a), the KEGG pathways were sorted by their nominal count values, mainly involving proteoglycans in cancer, pathways in cancer, estrogen signaling pathway, and other pathways. The most enriched pathway is proteoglycans in cancer, which contains 10 genes, followed by pathways in cancer, which contains 10 genes, and the estrogen signaling pathway contains 8 genes. According to the publications, the 3 components [36, 37] may play an important role in anti-tumor and estrogen signaling pathways.

In the GO function analysis, a total of 177 biological processes were enriched. The results showed that the key targets are closely related to the regulation of biological functions such as the ERBB2 signaling pathway, positive regulation of nitric oxide biosynthetic process, phosphatidylinositol-mediated signaling, and protein autophosphorylation. Regarding biological processes, a total of 8 processes were enriched with *P* < 0.01 and count ≥5, which were mainly related to signal transduction, negative regulation of the apoptotic process, cell proliferation, and positive regulation of transcription from RNA polymerase II promoter. In cell components, it was mostly related to the nucleus, cytosol, plasma membrane, and cytoplasm. Among the molecular function, *P* < 0.01 and count ≥5 were enriched in a total of 4 biological processes, which were related to protein binding and ATP binding, enzyme binding, and identical protein binding. See Figure 8(b) for details.

A network relationship diagram of “components-core targets-important pathways” using Cytoscape software visualization analysis was constructed. As shown in Figure 8(d), this network contains 31 nodes (3 chemical components, 15 target points, and 13 related pathways) and 115 edges (the relationships between them). The larger the point in the figure is, the more important the position in the network is. Marein, flavanomarein, and okanin mainly acted on 15 targets such as EGFR, AKT1, SRC, HRAS, and CASP3; the detailed related topology information is shown in Table 5. The targets involve proteoglycans in cancer, pathways in cancer, estrogen signaling pathway, Ras signaling pathway, and others. The targets exert anti-tumor and anti-inflammatory effects, which are consistent with pharmacological research on the activity of marein.

Marein and its metabolites may act on the same target to exert a similar activity. The intersection of the core targets of the three components shows that they may act on the six common targets GSTP1, PTPN1, SRC, ME2, HMGCR, and CASP3, and SRC are the core targets in the whole network. SRC; a proto-oncogene that is a member of the protein tyrosine kinase family, is one of the key factors leading to the increase of malignancy in many tumors [38].

## 4. Conclusion

A rapid and reliable UPLC-Q-TOF-MS/MS method was established for the first time to identify the metabolites of marein *in vivo*. The results remind us that when studying the efficacy and toxicity of flavonoids, the actual form of the flavonoids *in vivo* must be considered. The component-target-pathway network of marein, its aglycones, and flavanomarein was further established to illustrate their activity, and we found that marein and its metabolites act on key targets of EGFR, AKT1, SRC, HRAS, CASP3, and so on. It mainly involves pathways such as proteoglycans in cancer, pathways in cancer, estrogen signaling pathway, and Ras signaling pathway. The study provides a scientific basis for future research on the route of administration of flavonoids, the mechanism of action, and the discovery of lead compounds from active metabolites.

## Figures and Tables

**Table 1:** Characterization of metabolites after oral administration of marein in plasma and urine.

No.	t (min)	Meas. *m/z*	Pred. *m/z*	Formula	Ion	Diff. (ppm)	Fragment ions	Metabolite	Location
M1	5.62	495.0775	495.0780	C_21_H_20_O_14_	[M-H]−	−0.97	151.0034, 135.0426	M-2H + 3OH	P
M2	6.37	495.0779	495.0708	C_21_H_20_O_14_	[M-H]^−^	−0.16	301.0352, 151.0022, 135.0445	M-2H + 3OH	P
M3	5.65	479.0818	479.0831	C_21_H_20_O_13_	[M-H]^−^	−2.58	151.0031, 269.0442, 135.0438	M-2H + 2OH	P
M4	6.34	479.0817	479.0831	C_21_H_20_O_13_	[M-H]^−^	−2.93	151.0033, 135.0438	M-2H + 2OH	P
M5^*∗*^	5.58	463.0884	463.0882	C_21_H_20_O_12_	[M-H]^−^	0.50	287.0553, 269.0446, 151.0028, 135.0443	M-2H + OH	P, U
M6	5.99	463.0877	463.0882	C_21_H_20_O_12_	[M-H]^−^	−0.97	301.0709, 287.0552, 269.0445, 151.0028, 135.0444	M-2H + OH	P, U
M7	7.17	463.0878	463.0882	C_21_H_20_O_12_	[M-H]^−^	−0.80	301.0711, 287.0546, 269.0444, 151.0028, 135.0443	M-2H + OH	P, U
M8	7.55	463.0879	463.0882	C_21_H_20_O_12_	[M-H]^−^	−0.63	301.0711, 287.0546, 269.0440, 151.0028, 135.0443	M-2H + OH	P
M9	7.85	463.0873	463.0882	C_21_H_20_O_12_	[M-H]^−^	−1.77	301.0713, 287.0511, 269.0443, 151.0027, 135.0443	M-2H + OH	P
M10	8.13	463.0878	463.0882	C_21_H_20_O_12_	[M-H]^−^	−0.73	287.0548, 269.0452, 151.0026, 135.0442	M-2H + OH	P
M11	8.47	479.1205	479.1195	C_22_H_22_O_12_	[M-H]^−^	2.13	303.0871, 269.0457, 151.0028	M + OH + CH_3_	U
M12	9.27	479.1209	479.1195	C_22_H_22_O_12_	[M-H]^−^	3.11	303.0870, 151.0027, 135.0443	M + OH + CH_3_	U
M13^*∗*^	7.32	447.0930	447.0932	C_21_H_20_O_11_	[M-H]^−^	−0.44	285.0395, 151.0025, 135.0444	M-2H	P
M14	7.52	491.1194	491.1195	C_23_H_24_O_12_	[M-H]^−^	−0.06	315.0866, 151.0028, 135.0442	M + CH_3_CO	P, U
M15	7.94	491.1188	491.1195	C_23_H_24_O_12_	[M-H]^−^	−1.26	315.0865, 269.0443, 151.0021, 135.0441	M + CH_3_CO	P, U
M16	8.13	491.1197	491.1195	C_23_H_24_O_12_	[M-H]^−^	0.59	315.0860, 151.0027, 135.0446	M + CH_3_CO	U
M17	9.33	491.1190	491.1195	C_23_H_24_O_12_	[M-H]^−^	−0.98	315.0863, 151.0029, 135.0445	M + CH_3_CO	P, U
M18	9.77	491.1191	491.1195	C_23_H_24_O_12_	[M-H]^−^	−0.79	315.0869, 151.0030, 135.0443	M + CH_3_CO	P, U
M19	10.26	491.1193	491.1195	C_23_H_24_O_12_	[M-H]^−^	−0.31	315.0866, 151.0027, 135.0444	M + CH_3_CO	P, U
M20	6.36	641.1367	641.1359	C_27_H_30_O_18_	[M-H]^−^	1.19	465.1026, 303.0857, 151.0026	M + OH + CH_3_ + GluA	U
M21	6.74	641.1337	641.1359	C_27_H_30_O_18_	[M-H]^−^	−2.22	465.1017, 303.0866, 151.0028, 135.0442	M + OH + CH_3_ + GluA	U
M22^*∗*^	5.07	625.1410	625.1410	C_27_H_30_O_17_	[M-H]^−^	0.03	449.1087, 287.0551, 151.0023, 135.0448	M + GluA	P
M23^*∗*^	6.73	625.1410	625.1410	C_27_H_30_O_17_	[M-H]^−^	−0.01	449.1058, 287.0553, 269.0461, 151.0025, 135.0447	M + GluA	P
M24^*∗*^	9.15	287.0552	287.0561	C_15_H_12_O_6_	[M-H]^−^	−3.14	135.0447	M-GluA	P
M25	6.16	477.1034	477.1038	C_22_H_22_O_12_	[M-H]^−^	−0.75	301.0706, 151.0027, 135.0442	M-2H + OH + CH_3_	P, U
M26^*∗*^	6.80	477.1035	477.1038	C_22_H_22_O_12_	[M-H]^−^	−0.63	301.0708, 151.0029, 135.0444	M-2H + OH + CH_3_	P, U
M27^*∗*^	7.46	477.1036	477.1038	C_22_H_22_O_12_	[M-H]^−^	−0.38	301.0710, 151.0028, 135.0452	M-2H + OH + CH_3_	P, U
M28^*∗*^	8.56	477.1038	477.1038	C_22_H_22_O_12_	[M-H]^−^	0.00	301.0706, 151.0026, 135.0443	M-2H + OH + CH_3_	P, U
M29	9.07	477.1038	477.1038	C_22_H_22_O_12_	[M-H]^−^	−0.10	301.0708, 151.0027, 135.0442	M-2H + OH + CH_3_	P, U
M30^*∗*^	9.70	477.1045	477.1038	C_22_H_22_O_12_	[M-H]^−^	1.36	301.0714, 151.0028, 135.0479	M-2H + OH + CH_3_	P, U

*Note.* M: marein; P: plasma; U: urine; and ^*∗*^common metabolites for both modes of administration.

**Table 2:** Characterization of metabolites after injection of marein in plasma and urine.

No.	t (min)	Meas. *m/z*	Pred. *m/z*	Formula	Ion	Diff. (ppm)	Fragment ions	Metabolite	Location
M′0	7.52	449.1090	449.1089	C_21_H_22_O_11_	[M-H]^−^	2.66	287.0523, 269.0458, 151.0034, 135.0448	M′	P, U
M′1	5.67	449.1101	449.1089	C_21_H_22_O_11_	[M-H]^−^	3.13	287.0566, 269.0458, 151.0035, 135.0451	M′-2H + 2H	P, U
M′2	6.51	545.0620	545.0606	C_21_H_22_O_15_S	[M-H]^−^	2.51	269.0435, 151.0064	M′ + OH + SO_3_	P, U
M′3	7.38	545.0624	545.0606	C_21_H_22_O_15_S	[M-H]^−^	3.35	135.0404	M′ + OH + SO_3_	P, U
M′4	5.35	529.0683	529.0657	C_21_H_22_O_14_S	[M-H]^−^	4.91	449.1099, 287.0565, 269.0458, 151.0034	M′ + SO_3_	P, U
M′5	6.94	529.0680	529.0657	C_21_H_22_O_14_S	[M-H]^−^	4.31	449.1101, 287.0565, 269.0458, 151.0034	M′ + SO_3_	P, U
M′6^*∗*^	5.68	463.0898	463.0882	C_21_H_20_O_12_	[M-H]^−^	3.50	301.0706, 287.0548, 151.0044	M′-2H + OH	P
M′7	7.38	463.0876	463.0882	C_21_H_20_O_12_	[M-H]^−^	−1.27	287.0551, 151.0029	M′-2H + OH	P
M′8	5.70	447.0943	447.0932	C_21_H_20_O_11_	[M-H]^−^	2.45	287.0536, 269.0446	M′-2H	P
M′9	6.18	447.0942	447.0932	C_21_H_20_O_11_	[M-H]^−^	2.16	151.0028, 269.0459	M′-2H	P
M′10^*∗*^	7.38	447.0947	447.0932	C_21_H_20_O_11_	[M-H]^−^	3.17	285.0415, 267.0301, 151.0034, 135.0448	M′-2H	P, U
M′11	8.03	433.1129	433.1140	C_21_H_22_O_10_	[M-H]^−^	−2.56	257.0812, 151.0404, 135.0444	M′-OH	U
M′12	8.43	433.1135	433.1140	C_21_H_22_O_10_	[M-H]^−^	−1.04	257.0812, 135.0444	M′-OH	U
M′13	9.74	433.1151	433.1140	C_21_H_22_O_10_	[M-H]^−^	2.65	257.0821	M′-OH	P, U
M′14	6.86	463.1241	463.1245	C_21_H_22_O_10_	[M-H]^−^	3.47	301.0721, 151.0034, 135.0448	M′ + CH_3_	P, U
M′15	7.23	463.1257	463.1245	C_21_H_22_O_10_	[M-H]^−^	2.58	301.0709, 151.0027, 135.0443	M′ + CH_3_	P, U
M′16	9.25	463.1243	463.1245	C_21_H_22_O_10_	[M-H]^−^	−0.62	301.0710, 151.0029, 135.0443	M′ + CH_3_	U
M′17	6.84	419.1346	419.1347	C_21_H_24_O_9_	[M-H]^−^	−0.35	135.0442	M′ + 2H-2OH	U
M′18^*∗*^	4.85	625.1429	625.1410	C_27_H_30_O_17_	[M-H]^−^	3.08	449.1105, 287.0561, 269.0453, 151.0035, 135.0448	M′ + GluA	P, U
M′19	5.20	625.1428	625.1410	C_27_H_30_O_17_	[M-H]^−^	2.91	463.0875, 287.0553, 151.0028, 135.0442	M′ + GluA	P, U
M′20	6.38	625.1414	625.1410	C_27_H_30_O_17_	[M-H]^−^	0.68	449.1089, 287.0548, 151.0027, 135.0438	M′+GluA	U
M′21^*∗*^	6.78	625.1430	625.1410	C_27_H_30_O_17_	[M-H]^−^	3.23	449.1098, 287.0567, 269.0448, 151.0034, 135.0452	M′ + GluA	P, U
M′22^*∗*^	9.28	287.0555	287.0561	C_15_H_12_O_6_	[M-H]^−^	−2.13	151.0051, 135.0436	M′-GluA	U
M′23^*∗*^	6.84	477.1051	477.1038	C_22_H_22_O_12_	[M-H]^−^	2.68	301.0711, 151.0028, 135.0446	M′-2H + OH + CH_3_	P, U
M′24^*∗*^	7.58	477.1036	477.1038	C_22_H_22_O_12_	[M-H]^−^	−0.50	301.0715, 151.0031, 135.0450	M′-2H + OH + CH_3_	U
M′25^*∗*^	8.64	477.1049	477.1038	C_22_H_22_O_12_	[M-H]^−^	2.31	301.079, 151.0034, 135.0452	M′-2H + OH + CH_3_	P, U
M′26	9.45	477.1054	477.1038	C_22_H_22_O_12_	[M-H]^−^	3.27	301.0723, 151.0033, 135.0449	M′-2H + OH + CH_3_	P
M′27^*∗*^	9.76	477.1052	477.1038	C_22_H_22_O_12_	[M-H]^−^	2.94	301.0709, 151.0029	M′-2H + OH + CH_3_	P
M′28	5.35	543.0829	543.0814	C_22_H_24_O_14_S	[M-H]^−^	2.76	135.0453	M' + CH_3_ + SO_3_	P, U
M′29	5.84	543.0832	543.0814	C_22_H_24_O_14_S	[M-H]^−^	3.44	287.0559, 151.0012, 135.0454	M′ + CH_3_ + SO_3_	P, U
M′30	6.92	543.0812	543.0814	C_22_H_24_O_14_S	[M-H]^−^	−0.26	287.0516, 135.0449	M′ + CH_3_ + SO_3_	U
M′31	7.42	543.0818	543.0814	C_22_H_24_O_14_S	[M-H]^−^	0.74	287.0547, 151.001, 135.0432	M′ + CH_3_ + SO_3_	U

*Note.* M′0: marein, P: plasma, U: urine, and ^*∗*^common metabolites for both modes of administration.

**Table 3:** The results of area%, Ln (area%), and the time of incubation of marein in rat liver microsomes.

t (min)	Area%	Ln (area%)
0	100	4.61
5	98.69	4.59
15	86.70	4.46
30	82.34	4.41
45	77.93	4.36
60	68.01	4.22
90	67.21	4.21
120	62.76	4.14

**Table 4:** The results of the half-time period, CL_int_, CL′_int_, and CL_H_ of marein in rats liver microsomes.

Parameter	Unit	SD rat
t_1/2_	min	177.6923
CL_int_	mL·(min·mg protein)^−1^	0.0065
CL′_int_	mL·(min·kg)^−1^	11.7000
CL_H_	mL·(min·kg)^−1^	9.9382
ER	—	0.1506

**Table 5:** Relevant topological information of 15 key targets.

NO.	Uniprot ID	Gene name	Protein name	BC	CC	Degree
1	P12931	SRC	Proto-oncogene tyrosine-protein kinase SRC	0.0671	0.4160	34
2	P31749	AKT1	RAC-alpha serine/threonine-protein kinase	0.0971	0.4325	33
3	P00533	EGFR	Epidermal growth factor receptor	0.0702	0.4067	31
4	P01112	HRAS	GTPase HRas	0.0680	0.4022	31
5	P07900	HSP90AA1	Heat shock protein HSP 90-alpha	0.0494	0.4000	29
6	P45983	MAPK8	Mitogen-activated protein kinase 8	0.0664	0.3978	29
7	P05019	IGF1	Insulin-like growth factor I	0.0669	0.4160	27
8	P61586	RHOA	Transforming protein RhoA	0.0379	0.3872	26
9	P29474	NOS3	Nitric oxide synthase	0.0775	0.4007	24
10	P10275	AR	Androgen receptor	0.0542	0.3949	23
11	P03372	ESR1	Estrogen receptor	0.0464	0.4090	22
12	P02768	ALB	Albumin	0.0796	0.4113	21
13	Q06124	PTPN11	Tyrosine-protein phosphatase nonreceptor type 11	0.0072	0.3765	21
14	P17612	PRKACA	cAMP-dependent protein kinase catalytic subunit alpha	0.0389	0.3664	21
15	P42574	CASP3	Caspase-3	0.0544	0.3956	20

## Data Availability

All data are included in the article.

## Abbreviations

*C. tinctoria*: *Coreopsis tinctoria* Nutt.
MIN6: Islet beta cells of MIN6 mice
HepG2: Human hepatocellular carcinomas
HIF-1*α*: Hypoxia-inducible factor-1
SGLT2: sodium-dependent glucose transporters 2
CMC-Na: Carboxymethylcellulose sodium
ESI: Electron spray ionization
CE: Collision energy
CL: Clearance rate
t_1/2_: Elimination half-life
PPI: Protein-protein interaction
KEGG: Kyoto encyclopedia of genes and genomes
GO: Gene ontology
DAVID: The database for annotation, visualization, and integrated discovery
LG: Liquiritigenin
ILG: Isoliquiritigenin
LQ: liquiritin
ILQ: Isoliquiritin
EGFR: Epidermal growth factor receptor
AKT1: Proto-oncogene c-Akt
SRC: Sarcoma gene
HRAS: Harvey-RAS
CASP3: Caspase 3
GSTP1: Glutathione S-transferase pi-1
PTPN1: Protein tyrosine phosphatase nonreceptor type 1
ME2: Malic enzyme 2
HMGCR: HMG-CoA reductase
BC: Betweenness centrality
CC: Closeness centrality

## Supplementary Materials

Supplementary Materials The graphical abstract of the article is given in the supplementary material.
