# Supplementary figures and images for: Integrated UPLC-Q-TOF-MS/MS and Network Pharmacology Approach to Investigating the Metabolic Profile of Marein of Coreopsis tinctoria Nutt
Source: Evid Based Complement Alternat Med. 2022 May 23;2022:6707811. doi: 10.1155/2022/6707811 (PMC9152369; doi:10.1155/2022/6707811)

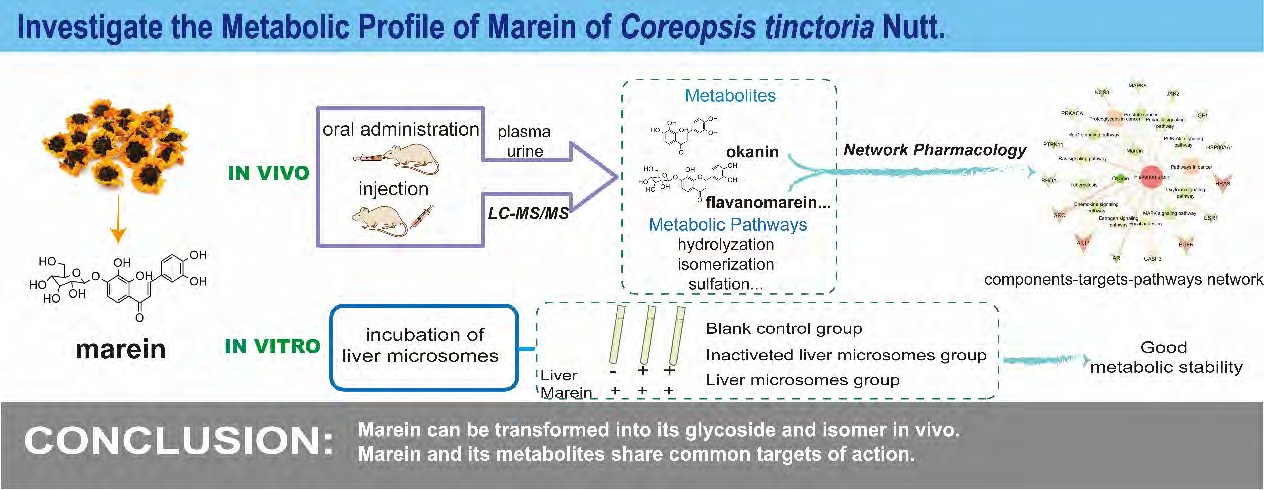


1. Graphical abstract

Supplement: Supplementary Materials — The graphical abstract of the article is given in the supplementary material. [file 6707811.f1.docx]
